# Prevalence and Incidence of Abnormal Behaviours in Individually Housed Sheep

**DOI:** 10.3390/ani2010027

**Published:** 2012-02-06

**Authors:** Mariko Lauber, Judy A. Nash, Allan Gatt, Paul H. Hemsworth

**Affiliations:** 1 Animal Welfare Science Centre, Melbourne School of Land and Environment, University of Melbourne, Parkville, Victoria 3010, Australia; E-Mails: janash@unimelb.edu.au (J.A.N); a.gatt1986@hotmail.com (A.G); phh@unimelb.edu.au (P.H.H); 2 Animal Welfare Science Centre, The Department of Primary Industries, 600 Sneydes Road, Werribee, Victoria 3030, Australia

**Keywords:** abnormal behaviour, individual housing, sheep, time budgets

## Abstract

**Simple Summary:**

Concern has been raised in Australia about the welfare of individually penned sheep housed indoors. This study examined the prevalence and incidence of abnormal behaviours in 96 individually housed sheep. Almost three quarters of the sheep displayed one or more of the behaviours of pacing, and chewing and nosing pen fixtures for more than 10% of the day. The prevalence and incidence of these ‘abnormal’ behaviours appears high, but without a comprehensive appreciation of other aspects of the animal’s biology, such as stress physiology and fitness characteristics, it’s difficult to understand the welfare implications of these behaviours.

**Abstract:**

This study examined the prevalence and incidence of abnormal behaviour in sheep housed individually indoors. Ninety-six castrated Merino sheep were observed using 15-min instantaneous sampling between 08:15 and 18:15 h for two consecutive days over a 3-week period. Sheep on average spent 62% of their time idle, 17% feeding, 1% drinking, 5% pacing, 10% chewing pen fixtures and 4% nosing pen fixtures. Pacing behaviour was predominantly seen in the morning with sheep on average spending 14% of their time pacing. Sheep on average spent 4% of their time in the morning and 13% of their time in the afternoon chewing pen fixtures. In the afternoon, the predominant behaviour was idle with sheep on average spending 71% of their time idle. Seventy-one percent of the sheep displayed one or more of the behaviours of pacing, and chewing and nosing pen fixtures for more than 10% of the day and 47% displayed one or more of these behaviours for more than 20% of the day. The prevalence and incidence of these ‘abnormal’ behaviours appears high, especially in relation to that of sheep grazed outdoors on pasture, and raises the question of the welfare risk to these animals. However, without a more comprehensive appreciation of other aspects of the animal’s biology, such as stress physiology and fitness characteristics, it is difficult to understand the welfare implications of these behaviours.

## 1. Introduction

There are approximately 40 farms housing ultra fine wool sheep in Australia involving an estimated 25,000 sheep [1]. Sheep in some of these systems are housed individually and concern has been expressed that sheep individually housed indoors show abnormal oral behaviours and/or stereotypical behaviour [2]. In this paper, we use the term ‘abnormal behaviour’ as shorthand to include behaviours such as stereotypic, displacement and redirected behaviours. In comparison to sheep grazed on pasture, anecdotal observations by the authors indicate a high prevalence of the behaviours of pacing and interactions with pen fixtures in individually housed sheep; therefore, this study examined the prevalence of abnormal behaviours in individually, indoor housed sheep.

The use of the term abnormal behaviour in domestic animals invariably raises questions about what is normal [3], particularly when most behavioural differences between wild and domestic animals appear to be quantitative rather than qualitative in character, and best explained in differences in response thresholds [4]. Considered as an aspect of the behaviour of an animal, abnormal behaviour is frequently defined as behaviour that is either atypical for the species, outside the normal behaviour pattern that has evolved in the natural habitats of the species or outside the range usually observed in the species in non-captive situations [5]. It has been proposed that the welfare of the animal is at risk if stereotypies occur for 10% of the animal's waking life [6] and if they occur in more than 5% of all animals [7]. Suffering and maladaptiveness are criteria often used when referring to abnormality of behaviour [4,8].

While research has led to a better understanding of the causation of some abnormal behaviours in animals in captivity, the function and, thus, adaptive significance of abnormal behaviours are often poorly understood. For example, the stereotypies of bar-biting and vacuum chewing (champing) in stalled or tethered sows may be a conditioned response arising from restricted feed intake [9], while wool-biting in confined sheep may be a redirected foraging behaviour arising from reduced foraging opportunities [10]. Therefore, in this study the range of behaviours labeled as ‘abnormal’ are behaviours that are commonly seen in sheep, but are regarded as abnormal behaviours when their prevalence and incidence exceed what is seen in an extensive setting.

There is limited research on the behaviour of adult wool-producing sheep individually housed indoors. Yurtman *et al.* [11] examined the impact of daily protein intake on abnormal behaviours of energy-restricted, individually penned, lambs undergoing fattening. They found that increasing crude protein content, from 148.6 to 214.9 g/kg DM per day, significantly increased oral stereotypies, particularly bar biting of feed hoppers. Vasseur *et al.* [10] examined the effect of supplementing the concentrate diet with fibre (straw) on abnormal behaviour in indoor group-housed sheep. They found that providing dietary fibre significantly reduced wool biting or wool damage. They also found that increasing the frequency of feeding did not affect wool biting or wool damage. Furthermore, wool biting in general increased with time confined. However, it is important to note that the sheep used in this study were introduced to the indoor housing system from a pasture based system only 1 week prior to the 15-week study and had not previously been housed indoors. Copper and Jackson [12] compared the time budgets of behaviour of experimental sheep intensively housed on straw and fed hay and a concentrate ration with sheep housed on wooden slats and fed only concentrates. They found that the sheep housed on straw spent more time lying, ruminating and moving than the sheep on slatted floors. Immediately after being fed their concentrate ration, sheep on the slatted floor showed a number of redirected oral activities including bar biting, slat licking, wool eating and repetitive licking after eating concentrates. Overall, sheep on slats spent 43% of their time displaying redirected oral behaviours, whereas sheep on straw were not observed displaying these behaviours. These three studies indicate that sheep housed indoors and fed concentrated diets can develop abnormal behaviours, such as wool biting and biting/chewing pen fixtures, and that protein and fibre content of diets can impact on the development of abnormal behaviours.

There appears to be increasing community concern about the welfare of domestic animals. Confinement housing of livestock appears to be at the forefront of these concerns. Studies of animal behaviour are important contributors in understanding the welfare of animals in captivity. The present study examined the prevalence and incidence of abnormal behaviours in commercial ultra-fine wool Merino sheep individually housed indoors.

## 2. Experimental Section

### 2.1. Subjects

Ninety-six ultra-fine merino wethers were studied in a 960-sheep indoor, individually housed, wool production system in Victoria, Australia for two consecutive days in autumn. The sheep had been housed in this facility for at least 6 mo. Forty-eight pairs, with animals in each pair housed adjacent, were randomly selected within three locations (blocks) of the facility. There was natural lighting in the facility provided by windows on three sides and temperature was not controlled.

Two types of pens housed the sheep: 1.42 m × 0.88 m (l × w) with feeders attached to the front of the pens and 1.52 m × 0.96 m (l × w) with feeders located within the pens. Pen walls were constructed of wooden horizontal bars so that sheep had the opportunity for visual and tactile contact with neighbours.

Sheep were fed once daily and depending on their size, daily received about 600 g of a diet consisting of lucerne hay, oaten hay and straw equivalent to about 4.4 ME KJ/day. Feeding, delivered by a stockperson from the corridor, commenced at 09:00 h and took about 2 h to complete. Water was available *ad libitum* via a trough in the back of each pen.

### 2.2. Behaviour Observations

Sixteen cameras were mounted on portable steel frames approximately 2.4 m above ground. The frames were attached to the pens one week prior to the commencement of recording to allow sheep to habituate to the apparatus. The cameras were installed 16–24 h prior to commencement of filming. Due to the limited availability of camera equipment, the 96 sheep were recorded in 3 separate blocks over 3 weeks (16 pairs per week).

Time budgets of behaviour, using the postures and behaviours listed in Table 1, were constructed from 15-min instantaneous samples taken from video recordings of the sheep between 08:15 and 18:15 h over a 2-day period. Although the cameras had IR capability, visibility at night was poor and therefore, only day-time measurements were taken. Video recordings were played and at each 15-min sample point, as per the time stamp on the video, the posture and behaviour displayed by each sheep were recorded. Instantaneous sampling is suitable for postures and behaviours of appreciable duration [13].

In addition to the instantaneous samples throughout the day, continuous observations were used to record the duration of postures and behaviours displayed by each sheep for the 30 min prior to feed being placed into their feed bin on each of the two observations days.

**Table 1:** Ethogram used in constructing time budgets.

**Postures**	
Standing	Standing stationary on four legs.
Lying	Lying on floor.
Mobile	Moving around in pen, not standing stationary. Not necessarily pacing
**Behaviours**	
Indistinguishable	Sheep visible but the animals behaviour was not clearly discernable (particularly when head was lowered)
Idle	Sheep clearly visible and not engaged in any of the following behaviours.
Feeding	Head lowered and directly in the feeder or floor where feed is visible
Drinking	Head lowered directly over water trough.
Walking	Walking in no distinct pattern.
Pacing	Walking but in a distinct pattern, such as frequent walking back and forth, weaving or moving in circles.
Chewing pen fixtures	Chewing pen fixtures (palings, floor slats, wire or feeder).
Nosing pen fixtures	Nosing or rubbing muzzle on pen fixtures (palings, floor slats, wire or feeder).
Head butt pen fixtures	Butting pen fixtures.
Pawing	Striking ground with forelegs.
Rearing	Forelegs on pen, back legs on ground, head raised.

### 2.3. Statistical Analysis

There were incomplete video data for 2 of the 96 sheep and thus data are presented on the 94 sheep which had complete video data.

For the observations conducted from 08:15 to 18:15 h in which instantaneous sampling was used, the proportion of 15-min sample points in which each animal displayed each posture or behaviour was calculated over the two days and, for convenience, these data are presented as percentage time that the behaviour was displayed over the two days. These behavioural data are also presented in two ways: the mean percentage of 15-min sample points in which sheep displayed each posture and behaviour are presented in both tabular and graphical forms and the distributions of the percentage of sample points in which each of the 94 sheep displayed the behaviours of pacing and interacting with the pen fixtures are presented as histograms.

For the data in which behaviour was continuously recorded for 30 min prior to feeding, the duration of each behaviour displayed by each animal was calculated. These data are presented as histograms showing the distributions of the percentage of time in the 30 min prior to feeding in which the 94 sheep displayed the behaviours of pacing and interacting with the pen fixtures. Pen type (1.42 m × 0.88 m with feeders attached to the front of the pens and 1.52 m × 0.96 m with feeders located within the pens) was distributed throughout the facility. Univariate General Linear Model (SPSS 16.0) was used to examine the effects of pen design on time spent displaying abnormal behaviours. To reduce skewness of the residuals, prior to the analysis, square root transformation was performed on the variable frequency of abnormal behaviours.

## 3. Results and Discussion

### 3.1. General Behaviour

**Figure 1:**
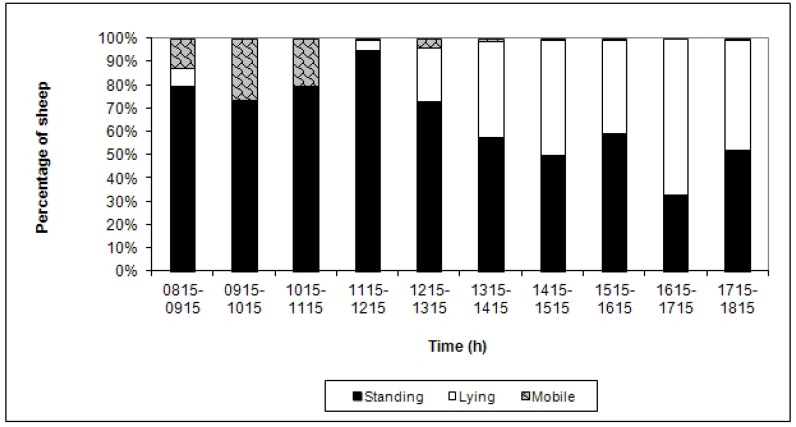
Mean percentage of sheep displaying the postures of standing, lying and mobile at each 15-min sample point during each h of observation over 2 days.

The mean percentage of 15-min sample points over the two days in which sheep displayed individual postures and behaviours is shown in Table 2. A high proportion of sheep were standing in the morning (Figure 1), with sheep on average spending 81% of their time standing (Table 2). Thus, the majority of sheep in the present study spent the morning standing or mobile, while the time spent lying was predominant in the afternoon.

**Table 2:** Mean percentage of 15-min sample points (from 08:15–18:15 h over the two days of observation) in which sheep displayed individual postures or behaviours. Standard errors of the mean are presented in parentheses.

	All Day	Afternoon	Morning
	(08:15–18:15 h)	(12:00–18:15 h)	(08:15–11:45 h)
**Postures**			
Standing	64.0 (1.2)	54.4 (1.5)	81.2 (1.8)
Lying	30.0 (1.0)	45.6 (1.5)	2.5 (0.5)
Mobile	6.3 (0.7)	0.6 (0.1)	16.3 (1.9)
**Behaviours**			
Indistinguishable	0.9 (0.1)	0.4 (0.1)	1.9 (0.3)
Idle	62.4 (1.3)	70.8 (1.6)	48.0 (1.9)
Feeding	16.6 (0.6)	9.4 (0.8)	29.4 (0.6)
Drink	1.3 (0.2)	1.8 (0.4)	0.6 (0.1)
Walking	1.0 (0.1)	0.4 (0.1)	2.2 (0.3)
Pacing	5.2 (0.7)	0.2 (0.1)	13.9 (1.9)
Chewing pen fixtures	9.7 (1.0)	12.7 (1.3)	3.7 (0.8)
Nosing pen fixtures	3.5 (0.3)	4.3 (0.4)	2.1 (0.3)
Head butt pen fixtures	0.4 (0.1)	0.4 (0.1)	0.3 (0.1)
Pawing	0.1 (0.0)	0.0 (0.0)	0.2 (0.1)
Rearing	0.1 (0.0)	0.0 (0.0)	0.1 (0.1)

### 3.2. Abnormal Behaviour

As shown in Table 2, the percentage of observations in which the three behaviours of pacing and chewing and nosing pen fixtures seems high and consequently these behaviours were labelled and combined as “abnormal behaviours”. There was no effect of pen type on the percentage of 15-min sample points over the two days in which abnormal behaviours were observed (F_1,92_ = 0.008, P = 0.929) and therefore, all data for both pen types were combined for further analysis. Pacing was predominantly seen in the morning with sheep on average spending 14% of their time pacing (Table 2). Sheep on average spent 4% of their time in the morning and 13% of their time in the afternoon chewing pen fixtures. The predominant behaviour in the afternoon was idle with sheep on average spending 71% of their time idle (Table 2).

**Figure 2:**
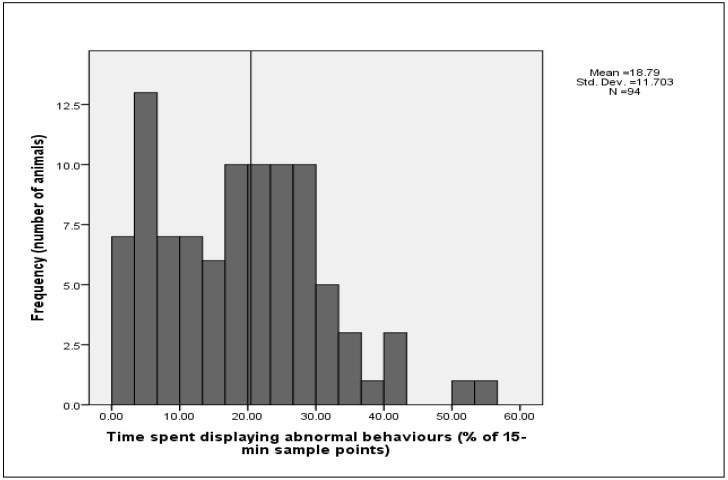
Histogram showing the distribution of time that sheep spent displaying abnormal behaviours (pacing and chewing and nosing pen fixtures) from 08:15–18:15 h over the two days of observations.

**Figure 3:**
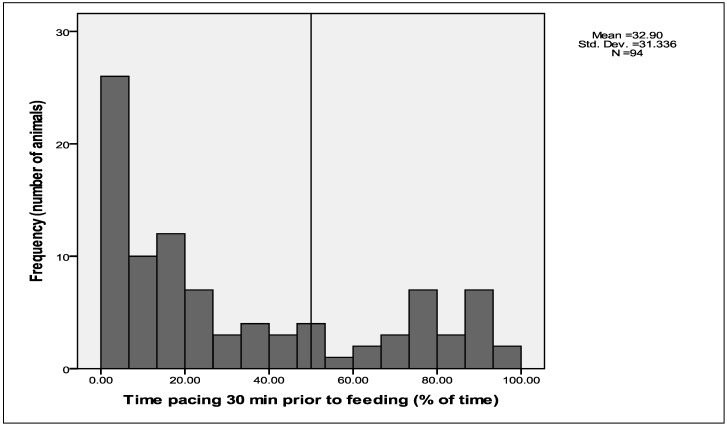
Histogram showing the distribution of time that sheep spent pacing in the 30 min prior to feeding.

Over the two observation days from 08:15 to 18:15 h, 67 sheep (71.3% of the sheep) displayed one or more abnormal behaviours for more than 10% of the time and 44 sheep (46.8%) displayed one or more of these abnormal behaviours for more than 20% of the time (Figure 2). While the display of abnormal behaviour was most pronounced before and after feeding, the data indicate that the abnormal behaviours were still prominent in the afternoon, well after feeding (Figure 3). Several authors have argued that the welfare of the animal is at risk if stereotypies occur for 10% of the animal's waking life [6] and if they occur in more than 5% of all animals [7]. Therefore, on this basis one could argue that the welfare of the sheep in this individual-housing system is compromised. However, this is premature without a closer analysis and consideration of the data.

The most frequent of these abnormal behaviours displayed immediately prior to feeding was pacing (Figure 3). Continuous observations for 30 min prior to feeding indicated that pacing was high for this period: mean time spent pacing was 32.9 (±SD of 31.3)%, with 46 sheep (47.9% of sheep) displaying pacing for more than 20% of the 30 min prior to feeding. There are examples in the literature of stereotypic pacing in captive animals that develop in association with food frustration [14].

**Figure 4:**
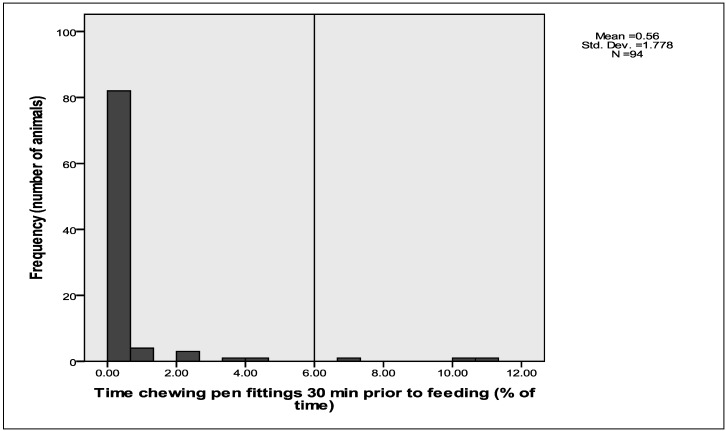
Histogram showing the distribution of time that sheep spent chewing pen fixtures in the 30 min prior to feeding.

In contrast, the duration of chewing pen fixtures prior to feeding, based on continuous observations for 30 min prior to feeding, was much less prominent than pacing: mean time spent chewing was 0.6% (±SD of 1.8) of the time with only 2 sheep (2.1% of sheep) displaying chewing for more than 5% of the 30 min prior to feeding (Figure 4). However, the incidence of chewing pen fittings continued throughout the day, unlike pacing which virtually ceased once feed was delivered to the sheep (Table 2). Done-Currie *et al.* [15] reported that individually housed sheep engaged in chewing pen fixtures, such as wire, bars and feeder. These behaviours along with other oral behaviours, such as chain rattling, occurred after feeding and during periods of relative quiet.

As discussed in the Introduction, there is evidence that the nutrition of sheep housed indoors can affect the development of abnormal behaviours, such as wool biting and chewing pen fixtures. Yurtman *et al.* [11] found increasing crude protein content significantly increased oral stereotypies, particularly bar biting of feed hoppers in energy-restricted, individually penned, lambs. Vasseur *et al.* [10] found that supplementing the concentrate diet with fibre, but not increasing the frequency of feeding, reduced wool biting in group-housed sheep. Similarly, Copper and Jackson [12] found that provision of straw on the floor and supplementing the concentrate diet with fibre reduced redirected oral activities such as bar biting, slat licking, wool eating and repetitive licking after eating concentrates.

Several authors have suggested that an inadequate opportunity to manipulate food or to forage may lead animals to redirect their motivation to feed or forage to manipulate other stimuli such pen fixtures, group-mates or themselves (captive giraffes [16], sheep [10], cattle [17], horses [18], parrots [19] and poultry [20]). Oral stereotypies have also been linked to diets high in carbohydrates as a possible way of generating extra saliva through chewing to help reduce the acidity of the gastric tract associated with these diets [16,18,21]. The sheep in the present study were fed once per day between 09:00 and 11:00 h and spent 16% on average during the day with their head in the feeder or close to the floor where feed was visible, presumably feeding. Stereotypies may develop in association with food frustration [14] and indeed some authors have proposed that oral stereotypies may develop from appetitive and redirected foraging behaviours [22]. Furthermore, in a review of stereotypies in captive animals, Mason [14] notes that stereotypic pacing often reaches a maximum just before feeding, while some oral stereotypies such sham-chewing, chain manipulation and drinker manipulation and polydipsia peak after feeding.

A number of authors have suggested [3] it is important that any interpretation of poor welfare is logically demonstrated and not inferred on the basis of the use of the term ‘abnormality’ of behaviour alone. The adaptive significance of stereotypies is controversial [23]. Stereotypies are generally most prevalent under conditions which are widely considered to be aversive to animals. However, there are examples where animals that perform high levels of stereotypies appear to cope better with poor environmental conditions [24,25].

Therefore, while it is generally agreed that environments that cause frustration and/or are particularly barren, often result in the development of abnormal behaviours such as stereotypies [26], directly equating the existence of abnormal behaviours with welfare risk is problematic. A more comprehensive understanding of their causation and corresponding effects on aspects of the animal’s biology, such as stress physiology and fitness characteristics including growth, injuries and health [27,28,29], will assist in assessing animal welfare implications. Alternatively, evidence, such as that shown by Cooper and Nicol [24] in bank voles, that the performance of abnormal behaviours may be associated with a change in perception and, therefore, may be a mechanism for coping with unfavourable conditions, may assist in assessing animal welfare. Nevertheless, it should be also recognised that the actual display of abnormal behaviours can cause harm to the animal, including physical injury, digestive complications and the loss of production due to the consumption of energy while eliciting the behaviour [11]. Furthermore, while the adaptive significance of abnormal behaviours such as stereotypies is controversial, their existence is indicative, at the least, of a past problem for the animal in coping with its conditions.

## 4. Conclusions

During the 2-day observation period, 71% of the observed sheep displayed one or more of the abnormal behaviours and 47% displayed one or more of abnormal behaviours for more than 20% of the day. The prevalence and incidence of these abnormal behaviours appears high, especially in relation to that of sheep grazed on pasture. Based on studies on sheep and other species, it is possible that an inadequate opportunity to manipulate food or to forage may lead sheep to redirect their motivation to feed or forage to other stimuli such as manipulating pen fixtures or to pace.

Without further research it is difficult to understand the welfare implications of these abnormal behaviours. The adaptive significance of abnormal behaviours is poorly understood; therefore, it is important that any interpretation of poor welfare is logically demonstrated and not inferred on the basis of the use of the term ‘abnormality’ of behaviour alone. Clearly further research on individually housed, ultra fine wool producing sheep is required to understand the causation and welfare implications of the abnormal behaviours observed in the present study.

## References

[1] Code of Practice for the Welfare of Sheep Housed for Wool Production. http://www.woolproducers.com.au/uploads/Final%20COP%20Housed%20Sheep%20Version%206.pdf.

[2] (2006). Intensive shedding of sheep for ultra fine wool: Why RSPCA wants this industry regulated. RSPCA Victoria News.

[3] Mills D.M., Mills D.M., Marchant-Forde J.N., Morton D.B., Phillips C.J.C., McGreevy P.D., Nicol C.J., Sandoe P., Swaisgood R.R. (2010). Abnormal/abnormality. The Encyclopaedia of Applied Animal Behaviour and Welfare.

[4] Price E.O. (2003). Animal Domestication and Behavior.

[5] Keeling L., Jensen P., Jensen P. (2009). Abnormal behaviour, stress and welfare. The Ethology of Domestic Animals.

[6] Broom D.M., Smith D. (1983). Stereotypies as animal welfare indicators. Indicators Relevant to Farm Animal Welfare.

[7] Wiepkema P.R., Smith D. (1983). On the significance of ethological criteria for the assessment of animal welfare. Indicators Relevant to Farm Animal Welfare.

[8] Seligman M.E.P., Walker E.F., Rosenham D.L. (2000). Abnormal Psychology.

[9] Lawrence A.B., Terlouw E.M.C. (1993). A review of behavioural factors involved in the development and continued performance of stereotypic behaviours in pigs. J. Anim. Sci..

[10] Vasseur S., Paull D.R., Atkinson S.J., Colditz I.G., Fisher A.D. (2006). Effects of dietary fibre and feeding frequency on wool biting and aggressive behaviours in housed Merino Sheep. Aust. J. Exp. Agr..

[11] Yurtman I.Y., Savas T., Karaagac F., Coskuntuna L. (2002). Effects of daily protein intake on the oral stereotypic behaviours in energy restricted lambs. Appl. Anim. Behav. Sci..

[12] Cooper J., Jackson R. (1996). A comparison of the feeding behaviour of sheep in straw yards and on slats (abstract). Appl. Anim. Behav. Sci..

[13] Martin P., Bateson P. (2007). Measuring Behaviour. An Introductory Guide.

[14] Mason G.J. (1991). Stereotypies: A critical review. Anim. Behav..

[15] Done-Currie J.R., Hecker J.F., Wodzicka-Tomaszewska M. (1984). Behaviour of sheep transferred from pasture to an animal house. Appl. Anim. Behav. Sci..

[16] Fernandez L.T., Bashaw M.J., Sator R.L., Bouwen N.R., Maki T.S. (2008). Tongue Twisters: Feeding enrichment to reduce oral stereotypy in Giraffe. Zoo Biol..

[17] Lindstrom T., Redbo I. (2000). Effect of feeding duration and rumen fill on behaviour in dairy cows. Appl. Anim. Behav. Sci..

[18] Waters A.J., Nicol C.J., French N.P. (2002). Factors influencing the development of stereotypic and redirected behaviours in young horses: Finding of a four year prospective epidemiological study. Equine Vet. J..

[19] Lumeij J.T., Hommers C.J. (2008). Foraging ‘enrichment’ as treatment for Pterotillomania. Appl. Anim. Behav. Sci..

[20] Blokhuis H.J., van der Haar J.W. (1989). Effects of floor type during rearing and of beak trimming on ground pecking and feather pecking in laying hens. Appl. Anim. Behav. Sci..

[21] Nicol C. (1999). Understanding equine stereotypies. Equine Vet. J..

[22] Wurbel H., Mills D.M., Marchant-Forde J.N., Morton D.B., Phillips C.J.C., McGreevy P.D., Nicol C.J., Sandoe P., Swaisgood R.R. (2010). Stereotypies. The Encyclopaedia of Applied Animal Behaviour and Welfare.

[23] Olsson I.A.S., Wurbell H., Mench J.A., Appleby M.C., Mench J.A., Olsson I.A.S., Hughes B.O. (2011). Behaviour. Animal Welfare.

[24] Cooper J.J., Nicol C.J. (1991). Stereotypic behavior affects environmental preference in bank voles, *Clethrionmys glareolus*. Anim. Behav..

[25] Mason G.J., Latham N.R. (2004). Can’t stop, won’t stop: Is stereotypy a reliable animal welfare indicator?. Anim. Welfare.

[26] Whemelsfelder F., Lawrence A.B., Rushen J. (1993). The concept of animal boredom and its relationship to stereotyped behaviour. Stereotypic Animal Behaviour: Fundamentals and Applications to Welfare.

[27] Broom D.M., Johnson K.G. (1993). Stress and Animal Welfare.

[28] Mellor D.J., Stafford K.J. (2000). Acute castration and/or tailing distress and its alleviation in lambs. New. Zeal. Vet. J..

[29] Barnett J.L., Hemsworth P.H. (2003). Science and its application in assessing the welfare of laying hens. Aust. Vet. J..

